# Western and non-western leadership styles and employee wellbeing: a case of a high-power distance context

**DOI:** 10.3389/fpsyg.2023.1261893

**Published:** 2024-01-19

**Authors:** Mats Ehrnrooth, Alexei Koveshnikov, Evgeniya Balabanova, Heidi Wechtler

**Affiliations:** ^1^Hanken School of Economics, Helsinki, Finland; ^2^Aalto University School of Business, Aalto, Finland; ^3^National Research University – Higher School of Economics, Moscow, Russia; ^4^Newcastle Business School, Callaghan, NSW, Australia

**Keywords:** transformational leadership, authoritarian leadership, paternalistic leadership, power distance, emotional exhaustion, Russia

## Abstract

The study combines an emic and etic perspective to test the relationships between three different (Western and non-Western) leadership styles, that is, transformational, authoritarian, and benevolent paternalistic, and follower emotional exhaustion in a high-power distance context of Russia. It employs hierarchical linear modeling (HLM) to analyse a sample of 403 followers to middle-level managers in Russian organizations. The analysis finds only transformational leadership to be generally negatively associated with emotional exhaustion. However, under conditions of high individual-level power distance orientation among followers, this association diminishes whereas that of authoritarian leadership and exhaustion increases. Benevolent paternalistic leadership is unrelated to emotional exhaustion. The study extends research on the relative importance of Western and non-Western leadership behaviors for employee wellbeing in high-power distance contexts and on how this importance differs across followers, thus highlighting the role of follower expectations in determining the effectiveness of leadership. It points toward the need for future research to simultaneously test the contingencies and relative importance of paternalistic, authoritarian, transformational, as well as other leadership styles in various cultures as well as to continue exploring the moderating influence of various cultural value orientations on these leadership styles’ follower effects.

## Introduction

The growing internationalization of business activities globally has motivated academics and practitioners to consider seriously the questions of effective leadership behaviors and their possible commonalities and differences across cultures (Dorfman et al., 1997; House et al., 2014; Crede et al., 2019). Moreover, an important question to ask is what competencies global leaders need to possess and what leadership behaviors need to be exhibited for effectiveness. On the one hand, whereas Western leadership styles such as transformational leadership (TL) have been portrayed largely positively in the context of Western countries and cultures, and their possible limitations in non-Western contexts have also been highlighted (see Blunt and Jones, 1997; Sanchez-Runde et al., 2011). On the other hand, more non-participative styles of leadership such as paternalistic (PL) and authoritarian (AL) leadership have been criticized as ineffective in the West but recently shown to be effective in high-power distance (PD) contexts, e.g., China and Russia (Cheng et al., 2004; Ma and Tsui, 2015; Koveshnikov et al., 2020) and/or their complex nature has been highlighted (e.g., Gunasekara et al., 2022).

An important indicator of a specific leadership behavior’s effectiveness is its impact on followers’ wellbeing (Barling and Frone, 2017; Harms et al., 2017; Walsh and Arnold, 2020). However, whereas in Western contexts leadership has been found to be “one of the most common sources” of stress (Skakon et al., 2010, p. 109), knowledge is still scarce about the specific relationships between various leadership styles and wellbeing in non-Western cultural contexts (Chu, 2014; Zhang et al., 2014; Wang et al., 2021). Moreover, more country-specific research on the effects of indigenous styles of leadership (e.g., Zhang et al., 2012; Patel et al., 2019) as well as more general research on the cross-cultural generalization of leadership behaviors (e.g., Liden, 2012; Crede et al., 2019) are continuously called for.

Therefore, our current understanding of the generalizability and the relative importance of different (Western and non-Western) leadership styles remains limited and needs to be examined, especially in non-Western cultural contexts. In the present study, we address the questions highlighted above by combining emic and etic perspectives (Lee et al., 2014) to analyse the relationships between three leadership styles and emotional exhaustion (EE) in the interesting but under-researched high-PD cultural context of Russia. Our first research question is formulated as follows: “What is the relative importance of TL, AL, and benevolent PL for follower EE in Russia?”

From an emic perspective, which prioritises “accounts, descriptions, and analyses expressed in terms of the conceptual schemes and categories regarded as meaning and appropriate by the native members of the culture whose beliefs and behaviors are being studied” (Lett, 1990, p. 130), we focus on Russia and examine the effects of authoritarian leadership (AL) and benevolent paternalistic leadership (PL) and the two leadership styles that can be seen as indigenous to Russia (Kets de Vries, 2000, 2001). In this way, we respond to continuous calls for more research on these two specific leadership styles (Chen et al., 2014; Harms et al., 2018) but also on leadership more generally, specifically in the still under-researched context of Russia (e.g., Balabanova et al., 2018). From an etic perspective, that prioritises “accounts, descriptions, and analyses expressed in terms of the conceptual schemes and categories regarded as meaning and appropriate by the community of scientific observers” (Lett, 1990, p. 130), we focus on the generalisability of the wellbeing effects of transformational leadership (TL), which was shown to generalize across a large number of cultures (e.g., Crede et al., 2019), to the high-PD Russian culture. In this way, we shed further light on the generalizability of TL to the Russian context where the effects of TL have not thus far been widely studied (for rare exceptions see Elenkov, 2002; Koveshnikov and Ehrnrooth, 2018) and, to the best of our knowledge, have not been studied at all in relation to employee wellbeing.

Additionally, in this study, we also examine possible moderating mechanisms and potential boundary conditions of the effects of each leadership style, as per recent call for more research “to understand the boundary conditions for… leadership” (Nielsen and Taris, 2019, p. 107), by focusing on individual-level power distance orientation (PDO). Therefore, our second research question is “Does PDO represent a boundary condition for the explanatory power of these nominal leadership theories, in support of contingency theories of leadership?” PDO is a cultural value which is particularly closely related to leadership (Kirkman et al., 2009). Yet, our current knowledge of its impact on TL effectiveness is inconclusive (Daniels and Greguras, 2014), and there is a lack of research on its impact on AL effectiveness (Harms et al., 2018). Therefore, this study addresses these gaps by examining how PDO moderates the relationships between the three focal leadership styles and EE in the context of Russia.

Outcome-wise, we focus on exploring the effects of the focal leadership styles on emotional exhaustion (EE) as a manifestation of employee wellbeing of wide-ranging societal and organizational importance (Hamstra et al., 2014; Liang, 2015). It “refers to feelings of strain, particularly chronic fatigue resulting from overtaxing work” (Fernet et al., 2012, p. 217) and reflects a specific form of employee (non-)wellbeing, the importance of which is emphasized by its relation to burnout (Maslach and Leiter, 2008).

Our study is based on a sample of 403 followers to middle-level managers in 232 domestic Russian organisations and offers three important contributions. First, we extend research on the generalizability and the relative importance of various (Western and non-Western) leadership styles (DeRue et al., 2011; Crede et al., 2019), thus providing ideas for what types of leadership behaviors may help global leaders succeed globally (Chatterjee et al., 2022). Second, by focusing on EE and the high-PD context of Russia, we extend research on leadership and wellbeing in non-Western cultural contexts (Chu, 2014; Zhang et al., 2014; Wang et al., 2021). Finally, we respond to calls for integrative research on how the influence of various leadership styles “differ across followers” (DeRue et al., 2011, p. 42; cf. Chen et al., 2014; Harms et al., 2017) and how PDO influences “the reactions of individuals to events or job characteristics that may increase wellbeing” (Daniels and Greguras, 2014, p. 1208).

## Context

In terms of efforts to understand the generalisability of theories of management and organization (Farh et al., 2007), the Russian context is under-studied. What we know is that AL and PL have played major, historically determined roles (Kets de Vries, 2000, 2001), that “autocratic” and “autonomous” leadership behaviors have been ranked the highest in Russia as compared to 61 other countries (Grachev et al., 2007, p. 821) and that the institutional conditions of doing business in Russia tend to reinforce authoritarian managerial styles in Russian organisations (Balabanova et al., 2018). At the same time, many scholars have argued that Russian leadership needs to move from the predominant paternalistic, authoritarian, and control-oriented leadership styles to more transformational ones (Elenkov, 2002; McCarthy et al., 2008, 2010). The context of Russia is highly relevant and important also given recent calls “for more research on the subject of autocratic leadership and authoritarian followers” (Harms et al., 2017).

## Hypotheses development

To understand how the three leadership styles individually and in combination with PDO influence EE among Russian followers, in what follows, we primarily draw on the focal leadership theories and the integrative Job Demands-Resources (JD-R) model of psychological stress and wellbeing (Bakker and Demerouti, 2007) to derive a set of hypotheses. The JD-R model posits that in every occupation, there are job-related factors, which can be classified as either job demands or job resources both with implications for work-related wellbeing. The former refers to those physical, psychological, social, or organizational aspects of the job, for instance, leadership, that require physical or psychological effort or skills to deal with it which may or may not negatively influence wellbeing. In contrast, job resources are those physical, psychological, social, or organizational aspects of the job that are functional in achieving job goals, reducing job demands, and stimulating personal growth, learning, and development. Below, we theorize the respective influences of the three focal leadership styles on followers’ EE.

### TL, resources and EE

*TL* comprises four related behavioral dimensions labeled core TL behavior or idealized influence, inspirational motivation, individualized consideration, and intellectual stimulation (e.g., Podsakoff et al., 1996). It is perhaps the most well-studied form of leadership (Avolio et al., 2009). In Western contexts, it has been found positively related to employee performance (Hamstra et al., 2014) and several aspects of follower wellbeing (Skakon et al., 2010; Harms et al., 2017). Transformational leaders are likely to offer good role models and meaningful (self)-leadership experiences to followers (through core TL behaviors) to instill self-confidence in followers’ ability to perform (through high inspirational motivation), delegate (through intellectual stimulation), and support employees (through individualized consideration). Thus, TL can be expected to improve follower wellbeing by the core mechanisms identified in the JD-R model, i.e., self-efficacy, job control, and support (Bakker et al., 2004; Bakker and Demerouti, 2007). In line with this, reviews and meta-analyses have concluded that TL decreases perceived strain, burnout, and work stress (Arnold, 2017) by reducing ambiguity, providing empowerment and support, increasing perceived organizational justice, and by allowing followers to use their resources more effectively (Harms et al., 2017). Thus, much theorizing and research in Western contexts suggests that TL reduces EE.

Yet, the enactment of TL is likely to differ across cultures and, for example, in hierarchical societies, it is likely to take more authoritarian, directive forms (e.g., Balabanova et al., 2018). It makes it important to control also for indigenous leadership styles when examining the effects of TL (Dickson et al., 2012). TL behaviors have been found to have significant main effects in relation to several outcomes in several cultural contexts (e.g., Avolio et al., 2009) albeit with rather inconclusive results and particularly so concerning employee wellbeing (Zhang et al., 2014). For instance, TL has been shown to have both weaker (e.g., Daniels and Greguras, 2014) and stronger (e.g., Crede et al., 2019) effects on employee outcomes in high power distance cultures.

Zooming in on the Russian context, by allocating more responsibility and accountability to followers, transformational leaders are likely to put followers under a different form of psychological pressure than what Russian employees are used to and desire (Kets de Vries, 2000; McCarthy et al., 2008). Russian followers are likely to feel more stressed than their Western counterparts from the expectation to take on initiative and bear responsibility for organizational processes and outcomes. This is then likely to make TL less effective among Russian employees as compared to employees in Western contexts. In this regard, previous research has pointed out that TL is not the prototypical or expected leadership style (cf. Kets de Vries, 2000; House et al., 2014). However, several extant studies show that—albeit the reservations above—TL affects positively Russian organizations (Elenkov, 2002) and employees (Balabanova et al., 2018; Koveshnikov and Ehrnrooth, 2018).

*H1:* In Russia, TL is negatively related to EE.

### AL, resources and EE

*AL* refers to “a leader’s behaviour of asserting strong authority and control over subordinates and demanding unquestioned obedience from them” (Chen et al., 2014, p. 799). It is much less researched compared to TL. However, in high-PD cultures, AL prevalence has been emphasised (Chen et al., 2014). The scarce extant research indicates that authoritarian leaders “may be expected to have employees who report lower levels of wellbeing” (Skakon et al., 2010). In their rare study on the consequences of AL for employee wellbeing in a Western setting, De Hoogh and Den Hartog (2009) found it to relate positively to burnout. Furthermore, research indicates that AL is negatively associated with favorable outcomes such as follower commitment, effort, and team solidarity across cultures (House et al., 2014, p. 253, 341). In line with this, previous studies found AL to be largely negatively related to organizational behaviors and attitudes in Taiwan (Chen et al., 2014) and Hong Kong (Sheer, 2010).

In some contrast to the above, one study conducted in Taiwan found AL to relate positively to follower attitudes, but only in the form of compliance and the leader-centric outcome of gratitude toward the leader (Cheng et al., 2004). Furthermore, Farh et al. (2006) noted that “authoritarian leaders may extract compliance from followers” but that they do so “through fear” and “their doing so has relational costs—lower satisfaction with supervision and lower organizational commitment” (p. 256). Overall, thus, both cross-cultural and local research suggest that AL has negative effects on employee wellbeing. However, Chen et al. (2014) found the influence of AL to be “particularly intriguing” in the Chinese setting (p. 814) and called for more research on the effects of AL. De Hoogh et al. (2015) provided some evidence of situational positive effects of AL on follower wellbeing in the form of psychological safety.

Building on the above, we expect that AL is likely to be more effective in Russia than in most other cultures because Russian employees tend to expect leaders to decide and not to delegate responsibility and accountability (McCarthy et al., 2008). In Russia, AL is likely to influence employee wellbeing positively by providing the key resources of support and feelings of self-efficacy. It is likely to do so not by empowering followers but exactly by the opposite, i.e., by making decisions for followers, by commanding and insisting that followers follow orders, and thus by leaving followers with limited responsibility. In these ways, Russian followers can be expected to feel less emotionally strained and psychologically stressed.

*H2:* In Russia, AL is negatively related to EE.

### Benevolent PL, resources and EE

Benevolent PL can be defined as leadership that takes “a personal interest in the workers’ off-the-job lives and attempt to promote workers’ personal welfare” (Pellegrini and Scandura, 2006, p. 267; cf. Chen et al., 2014). Similar to AL, PL is prevalent in high-PD cultures (Pellegrini and Scandura, 2006, 2008), but its effects are underexplored. Kets de Vries (2000) argued that “[p]aternalism can be a great source of strength because it makes for interdependence, security, and safety” (p. 78). Pellegrini and Scandura (2008) further noted that the effectiveness of benevolent PL may be underestimated. However, empirical research on the effects of PL is scarce (Aycan, 2006) albeit with a few notable exceptions in both Western (Pellegrini and Scandura, 2008) and non-Western cultures (Pellegrini et al., 2010; Chen et al., 2014). Overall, our current understanding of the effectiveness of PL remains limited (Pellegrini and Scandura, 2008, p. 567).

To complement this nascent literature, in this study, we focus on a particular form of PL which is benevolent PL. It involves an extended form of support and care for followers’ lives that is not only “distinct from authoritarianism” (Pellegrini and Scandura, 2008, p. 569) but also from TL (Pellegrini and Scandura, 2008, p. 575). Leaders exhibiting benevolent PL tend to promote workers’ “personal welfare” by—among other things—“taking a personal interest in the workers’ off-the-job lives” (Pellegrini and Scandura, 2006, p. 267; Pellegrini et al., 2010, p. 392). Such leaders act “like parents” and ensure “that the whole person is being attended to” in terms of both work-related and personal matters (Chen et al., 2014, p. 800). Consequently, benevolent PL was found to be positively associated with social support and organizational commitment (Farh et al., 2006).

Considering the above and in line with the JD-R model, benevolent PL is likely to relate negatively to follower EE, especially in high power distance cultures, such as Russia, where followers tend to expect leaders to be involved in followers’ personal lives (Pellegrini and Scandura, 2008) and to make important decisions on followers’ behalf (Kets de Vries, 2000). In support, it was noted that “Russians want and expect their leaders to take care of them” and such care is likely to reduce their work-related psychological stress and emotional strain (Kets de Vries, 2001, p. 617).

*H3:* In Russia, benevolent PL is negatively related to EE.

### Moderating effects of PDO

Many leadership theories suggest that *a fit* (cf. Pellegrini and Scandura, 2008) between leaders and followers is central to the effects of leadership. Such theories include several contingency theories of leadership, the *social identity theory of leadership* (Hogg, 2001), the importance of *congruence in expectations* (House et al., 2014; Zhang et al., 2014) and *congruence in preferences* (Daniels and Greguras, 2014), and generally *follower-centred leadership* theory (Uhl-Bien et al., 2014). We posit that individual-level PDO is a crucial factor that may influence the fit between leaders and followers and predetermine leaders’ effectiveness. *PDO* reflects the extent to which individual followers expect and accept “top–down direction from their leaders” (Kirkman et al., 2009, p. 746). Extant research suggests that it relates positively “to several indicators of wellbeing” (Daniels and Greguras, 2014, p. 1212).

Applying the fit argument to the three focal leadership styles, it is plausible that followers with high PDO are likely to be less receptive more generally to TL with its emphasis on follower participation and empowerment (Daniels and Greguras, 2014). In line with this, PDO was found to attenuate the positive effects of TL on, e.g., followers’ perceptions of procedural justice (Kirkman et al., 2009) and affective commitment (Newman and Butler, 2014). This suggests that PDO is also likely to attenuate the effects of TL on follower wellbeing.

Regarding AL, Cheng et al. (2004) found its relationship with leader-centric outcomes (follower compliance, gratitude toward and identification with the leader) to be positively moderated by follower authority orientation, which is closely related to PDO. However, Farh et al. (2006) studied whether follower traditionality orientation moderates the relationships between AL and several other outcomes but found only one moderation effect, again related to a leader-centric outcome (satisfaction with the leader). The relationships between AL and fear, compliance, identification, commitment, and gratitude were not moderated. It follows that extant research regarding the impact of power distance on the effectiveness of AL is inconclusive, in particular with respect to non-leader-centric outcomes such as wellbeing (Harms et al., 2018). However, De Hoogh et al. (2015) found that autocratic leadership is positively related to team psychological safety and performance among team members who accept hierarchy. This implies a moderation effect of PDO on the effects of autocratic leadership styles, including possibly also AL, with reference to employee wellbeing.

Finally, regarding benevolent PL, we expect that in the Russian culture where PL is a prototypical and widespread leadership style (Kets de Vries, 2000, 2001), its influences will be moderated by PDO in the same way as that of AL. Followers with high PDO can be expected to be more receptive to benevolent PL and such leadership style is likely to be more effective in reducing EE among high PDO followers.

In summary, the above suggests that the previously argued negative relationship between TL and EE will be weaker under conditions of high follower PDO, while the same condition will strengthen the corresponding negative relationships between AL and EE and between benevolent PL and EE.

*H4a:* In Russia, follower PDO moderates the negative relationship between TL and EE such that the relationship is weaker when PDO is high.

*H4b:* In Russia, follower PDO moderates the negative relationship between AL and EE such that the relationship is stronger when PDO is high.

*H4c:* In Russia, follower PDO moderates the negative relationship between benevolent PL and EE such that the relationship is stronger when PDO is high.

## Materials and methods

### Sample

The data were gathered using a telephone survey, which was administered by representatives of a Western professional data collection agency located in Russia. It surveyed white-collar employees working in large (with more than 500 employees), domestic organizations in Russia. These employees have been identified and selected by the data collection agency based on our objective to survey our study white-collar employees who are followers of middle-level managers. In this way, we wanted to ensure that our respondents were eligible to respond to our survey’s questions about their reactions as followers to their proximal leaders’ diverse leadership styles.

Altogether 967 white-collar employees were contacted, 403 agreed to participate in the survey (42% response rate). Position-wise, all were office employees with non-supervisory positions. The respondents came from 232 organisations located in Moscow and Saint-Petersburg and operating in five industries, namely, food processing, machine building, construction, metal, and banking. The average number of respondents per organization was 1.74, their average age was 36.3 (s.d. = 9.9), 35% of them were male, and the average tenure under the same supervisor was 4.5 (SD = 3.9). In total, 50% of the respondents were located in Moscow and 50% in Saint-Petersburg.

The original questionnaire was translated into Russian and then back translated into English by two different bilingual professional translators. Subsequently, the back-translated questionnaire items were first evaluated by the authors for correspondence with the original English language versions, and then the questionnaire was piloted on five native Russians. Several phrases that were identified as ambiguous have been clarified in the process.

### Measures

Except for employee gender, age, and tenure, all survey items were scored on a Likert response scale: for leadership items and PDO from 1 (*strongly disagree*) to 5 (*strongly agree*) and for job demands and EE from 1 (*never*) to 5 (*always*).

#### Dependent variable

*EE* was measured by three best-loading items in Maslach and Jackson’s (1981) original measurement instrument for EE except for the item ‘I feel burned out from my work’ which we excluded because we did not expect all employees to be familiar with the term ‘burned out’.

#### Independent variables

*TL* was measured by an abbreviated version of the measurement instrument developed by Podsakoff et al. (1996). We included nine best-loading (non-reverse coded) items based on the combined consideration of the construct domain and several validation studies (including Podsakoff et al., 1996): “core TL behaviors” (three items) and “high performance expectations,” “individualized consideration,” and “intellectual stimulation” (two items each). One item was excluded based on its poor loading on the latent factor.

*AL* was operationalized by four best-loading items in Sheer (2010) corresponding closely to the measures used by De Hoogh and Den Hartog (2009) and De Hoogh et al., (2015).

*Benevolent PL* was measured with five best-loading items in Pellegrini and Scandura (2006). One item was excluded based on its poor loading on the latent factor.

#### Moderating variable

*PDO* was measured with three items based on Farh et al. (2007) and Kirkman et al. (2009).

#### Control variables

In addition to controlling for organization by using it as the blocking variable in our HLM regression analyses, we also controlled for respondent gender, age, and tenure under the same supervisor. Gender was measured as a dichotomous categorical variable (Male = 0, Female = 1), age and tenure as the number of full years. We also controlled for *job demands* measured by three workload-related items from Bakker et al. (2004).

### Measurements quality

The measurement model fitted the data well as shown by the goodness-of-fit indices (*χ*^2^ = 743.585, df = 261, RMSEA = 0.056, GFI = 0.931). For each measure, reliability and validity were demonstrated. Cronbach’s alphas and composite reliabilities were superior to 0.70, average variance extracted was superior or close to 0.50, and bivariate correlations between the constructs were inferior to the square root of the average variance extracted, providing evidence that our measurements are reliable and valid.

### Common method variance

We first conducted Harman’s single-factor test. We conducted factor analysis that included all items in our model (i.e., associated with EE, TL, AL, PL, PDO, and job demands). Six factors with Eigenvalue’s superior to 1 were identified, and the first factor accounted for 22% of the total variance, substantially below the 50 percent threshold (Podsakoff et al., 2003). Second, considering that Harman’s test has been criticized, we also employed the unmeasured latent method construct (ULMC) method (e.g., Podsakoff et al., 2003). To do that, we performed a confirmatory analysis, where each item was associated with its theoretical latent factor and a method factor. Results indicated that the amount of variance associated with the method factor was 19%. As for the interpretation of the identified interactive effects in our study, prior research shows that common method variance is unlikely to inflate such effects (see Siemsen et al., 2010). Taking these results together, we can conclude that common method bias is not a serious threat to the interpretation of the results that follow.

## Results

Descriptive statistics and correlations between the constructs are presented in Table 1. For the analysis, all predictors except gender were grand mean centred, and the main predictors and the moderator were standardized to reduce possible multicollinearity problems. As our data are nested (i.e., collected from 232 organisations), we first evaluated the homogeneity of EE across organisations. The intra-class correlation was 0.16, indicating a relatively small homogeneity at the organizational level. In other words, the factors associated with EE are rather individual. Nevertheless, we used hierarchical linear modeling (HLM) to increase the accuracy of our estimations while testing our hypotheses. As our variables are measured using Likert scales, we graphically examined the shape of their distributions. In addition, skewness and kurtosis values helped to quantify the deviation from normality. No outliers were found, and normality indicators were within the accepted range (skewness ranging from −1 to +1, kurtosis ranging from −2 to +2).

**Table 1:** Means, standard deviations, and correlations.

	Mean	SD	1	2	3	4	5	6	7	8
1.Emotional exhaustion	2.363	1.138								
2.Transformational leadership	3.612	0.731	−0.110							
3.Authoritarian leadership	4.022	0.651	−0.153	0.321						
4.Benevolent paternalistic leadership	2.971	1.029	0.053	0.518	−0.001					
5.Power distance orientation	3.708	1.044	−0.325	0.195	0.389	−0.064				
6.Age	36.312	9.700	−0.036	0.062	−0.067	−0.003	0.050			
7.Gender (female = 1)	0.650	0.478	0.024	0.036	−0.029	0.059	0.031	−0.074		
8.Tenure with same supervisor	4.471	3.931	−0.163	0.095	−0.001	−0.068	0.094	0.561	−0.055	
9.Job Demands	3.250	1.093	0.642	−0.004	−0.042	0.078	−0.145	−0.041	−0.020	−0.113

*N* = 403. Correlations superior to |0.10| were significant at the 5% threshold and superior to |0.08| at the 10% threshold.

Estimates are presented in Table 2. Model 1 included the control variables only, Model 2 included the main effects (TL, AL, and PL), and in Model 3, we entered the moderator (PDO). Finally, Model 4 included, in addition, the three interactive terms.

**Table 2:** HLM results for emotional exhaustion.

	Model 1		Model 2		Model 3		Model 4	
	Estimate	SE	Estimate	SE	Estimate	SE	Estimate	SE
Intercept	2.363***	0.044	2.363***	0.043	2.363***	0.042	2.381***	0.045
**Controls**								
Gender (female)	0.039	0.044	0.037	0.044	0.048	0.042	0.049	0.042
Age	0.071	0.053	0.059	0.053	0.069	0.051	0.077	0.051
Tenure with same supervisor	−0.142**	0.053	−0.123*	0.053	−0.113*	0.052	−0.106*	0.051
Job Demands	0.718***	0.044	0.711***	0.044	0.684***	0.043	0.659***	0.044
**Main effects**								
Transformational Leadership (TL)			−0.107*	0.055	−0.102*	0.054	−0.039	0.054
Authoritarian Leadership (AL)			−0.105**	0.047	−0.023	0.048	−0.060	0.050
Benevolent Paternalistic Leadership (PL)			0.050	0.053	0.019	0.051	0.008	0.052
**Moderator**								
Power Distance Orientation (PDO)					−0.241***	0.047	−0.224***	0.046
**Interactions**								
TL x POD							0.142**	0.052
AL x PDO							−0.117**	0.046
PL x PDO							0.003	0.054
*R* ^2^	0.424		0.444		0.480		0.498	
*R*^2^ change	-		0.021**		0.036**		0.018**	

Two-tailed tests. * ≤ 0.05, ** ≤ 0.01, *** ≤ 0.001. Unstandardized coefficient estimates. *N* = 403 at the individual-level (*n* = 232 organizations).

In support of Hypothesis 1, TL was significantly and negatively related to EE (*b* = −0.107, *p* ≤ 0.05). In support of Hypothesis 2, AL was also significantly and negatively related to EE (*b* = −0.105, *p* ≤ 0.01). However, Hypothesis 3 was rejected as the relationship between EE and benevolent PL was non-significant (*b* = 0.050, ns). PDO was significantly and negatively related to EE (*b* = −0.241, *p* ≤ 0.001) so that the more power distance oriented a follower is, the less emotionally exhausted she/he feels (Model 3). This is logical in the Russian culture and generally in line with previous findings of positive relationships between power distance “and several indicators of wellbeing” (Daniels and Greguras, 2014, p. 1212).

**Figure 1:**
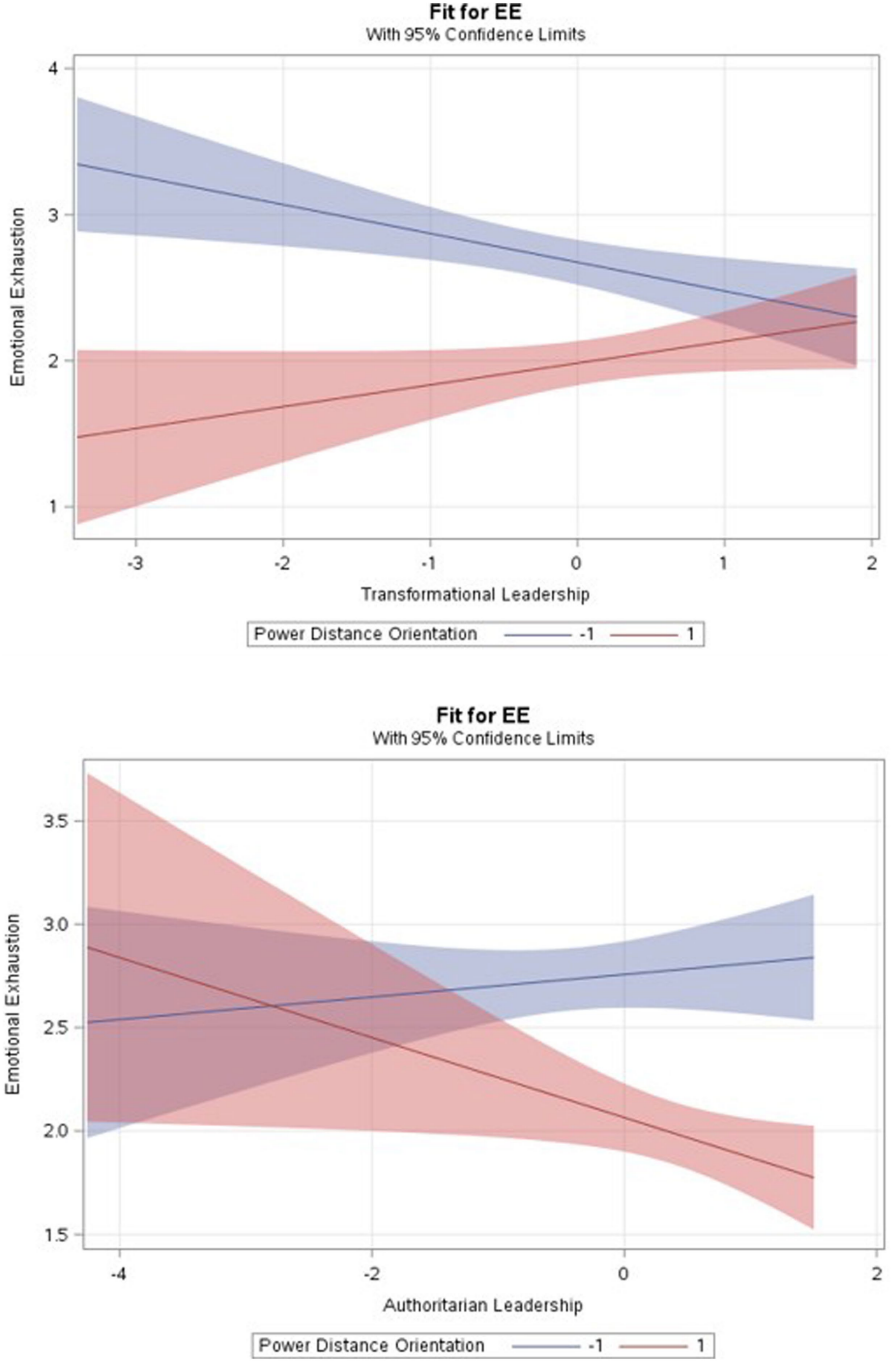
Interaction effects.

Model 4 shows that PDO moderated the relationship between TL and EE (*b* = 0.142, *p* ≤ 0.01) which means that PDO attenuates the relationship. Figure 1 illustrates the differential relationships under conditions of low and high PDO. To help the interpretation, Aiken and West’ test of simple slopes (Cohen et al., 2003) was conducted. It shows that the relationship is positive and significant when followers have high (+1 SD above the mean) PDO (*b* = 0.321, *p* ≤ 0.001) and that the relationship becomes negative and significant when followers have low (−1 SD above the mean) PDO (*b* = −0.369, *p* ≤ 0.001).

Model 4 also shows that the interactive terms together brought substantial additional variance (*R*^2^ change = 0.018, *p* ≤ 0.01) and that PDO negatively moderated the relationship between AL and EE (*b* = −0.117, *p* ≤ 0.01) which means that when followers have high PDO, the relationship between AL and EE is strengthened. When followers have high PDO, there is a negative relationship between AL and EE (*b* = −0.416, *p* ≤ 0.001) and vice versa (*b* = 0.276, *p* ≤ 0.001) (see Figure 1). Finally, no significant moderation effect of PDO and benevolent PL was found. Therefore, Hypotheses H4a and H4b were confirmed while H4c was rejected.

## Discussion

### Theoretical contributions

Our study makes several contributions. First, it contributes to the literature on the cross-cultural generalizability of various leadership styles and their relative importance for followers’ wellbeing in several respects. When it comes to TL, generally, the evidence for the cross-cultural generalisability of the effectiveness of TL has been somewhat mixed (Avolio et al., 2009; Kirkman et al., 2009; Crede et al., 2019). In this light, the present study supports the generalisation argument, specifically in relation to employee wellbeing (cf. Skakon et al., 2010; Harms et al., 2017); in that, we found TL to be negatively and significantly associated with EE. It implies that also in high-PD contexts, TL offers followers adequate psychological and emotional resources not only to perform well their work-related tasks (see Crede et al., 2019) but also to cope with emotional exhaustion.

Furthermore, previous research has found AL to have mainly negative effects on followers, e.g., in Taiwan (Chen et al., 2014), Hong Kong (Sheer, 2010), the Netherlands (De Hoogh and Den Hartog, 2009), and overall in 62 countries (House et al., 2014). The exceptions have mainly been leader-centric outcomes such as follower compliance (Cheng et al., 2004; Farh et al., 2006) and identification (Cheng et al., 2004) in high-PD contexts. It was even concluded that “authoritarianism … may be the least useful leadership behaviour” (Farh et al., 2006, p. 813). The significant and negative main effect of AL in our study goes against this conclusion. However, as discussed below, to understand the complex influence of AL on followers’ emotional exhaustion, we need to account for followers’ PDO. When we do so, the situation changes.

As for the effects of benevolent PL, our non-significant findings do not support several previous studies that found PL to influence followers positively by decreasing their exhaustion (Farh et al., 2006; Chen et al., 2014; cf. Pellegrini et al., 2010). However, none of the latter studies controlled for potentially “competing” forms of leadership, such as TL. When controlling for TL and AL, our findings suggest that benevolent PL is an ineffective form of leadership in the Russian context when it comes to diminishing employee EE which can be explained by its supposedly weak resource-based effects. We note that compared to Cheng et al. (2004) and Chen et al. (2014), the correlations are quite different in our sample from Russia where benevolent PL is not correlated with either AL or PDO. Thus, in some cultures, benevolent PL may still be an important leadership style in decreasing follower EE in particular when combined with TL behaviors (Aycan et al., 2013).

To sum up this contribution, our study extends research on the relative importance of various leadership styles (DeRue et al., 2011) for followers’ EE. It provides additional evidence of the earlier established importance of TL (Avolio et al., 2009) and its special significance in non-Western contexts (see Crede et al., 2019) in relation to followers’ EE. At the same time, it questions some of the earlier findings concerning the positive influence of PL on followers’ wellbeing (Chen et al., 2014) and the argument that AL is generally the least useful leadership style (Farh et al., 2006). Our analysis shows that when controlling for other leadership styles, PL might not be that resourceful to help followers cope with EE. In contrast, AL might be an effective leadership style, helping followers with high PDO to deal with EE. Then, we return to this point below.

Second, our study contributes to the extant literature on the role of followers’ individual-level cultural attributes in determining followers’ organizational behaviors and attitudes (Farh et al., 2006; Kirkman et al., 2009; Daniels and Greguras, 2014). It adds to our understanding of how followers’ PDO acts as a possible boundary condition for the effects of TL and AL on followers’ EE. Our finding that the negative relationship between TL and EE is attenuated (and in fact turns into a positive one) among followers with high PDO suggests that TL is effective only among already transformed followers, who have low PDOs. It appears that for high PDO followers, who are not used to a more delegating and empowering leadership, TL might be perceived as adding to their EE. Thus, followers’ PDO functions as a boundary condition for the effects of TL on followers’ EE. It indicates that indeed TL can be effective in high-power distance contexts (see also Crede et al., 2019), also improving followers’ wellbeing but only among followers with low PDO.

As for AL, our finding that AL appears to effectively decrease emotional exhaustion only among followers with high PDO points to the boundary conditioning role of followers’ PDO also to the effects of AL on followers’ EE. In contrast, we find that among followers with low PDO AL tends to increase EE. This provides a more nuanced understanding of the conditions under which AL is effective, and by doing so, our study extends recent findings of De Hoogh et al. (2015) by pointing out that AL may work in high power distance contexts and may have positive influence on followers but only among followers with high PDO (Harms et al., 2017). It appears that AL is a source of psychological stress for followers with low PDO.

To sum up our second contribution, our results extend research on how the influence of various leadership behaviors “differ across followers” (DeRue et al., 2011, p. 42; cf. Harms et al., 2017) and specifically research on “the reactions of individuals to events or job characteristics that may increase wellbeing” (Daniels and Greguras, 2014, p. 1208). In this way, our study points to the important role of follower expectations based on their individual-level PDO for successful leadership (House et al., 2014).

Finally, by focusing on Russia, our study also contributes to research on leadership and wellbeing in non-Western cultural contexts, which has thus far largely focused on China (e.g., Chu, 2014; Zhang et al., 2014; Wang et al., 2021). Past research has found Russian managers to believe that authoritative leaders, as opposed to authoritarian ones and closely reminiscent of transformational ones, are the most effective Russian leaders (McCarthy et al., 2010). In line with this, a few rare studies (see Elenkov, 2002; Koveshnikov and Ehrnrooth, 2018) offered some initial evidence that TL can be effective in Russia. The results of House et al. (2014) also support the contention that Russian followers, on average, desire more transformational-type leaders. While providing qualified evidence in support of this extant research, here with specific reference to employee wellbeing, our study suggests that in Russia this may apply only to followers with low PDO. For followers with high PDO, AL may be a better leadership style.

### Practical implications

The study offers additional contextual evidence for the importance of leaders taking individual-level cultural characteristics of followers into account and trying to adjust their leadership styles and/or find other ways to promote employee resources accordingly. This is true for leaders operating in the high-PD context of Russia when it comes to follower PDO and EE. However, our study also suggests that we need to qualify extant conclusions that leaders who do not comply with “societal expectations are almost certain to fail” (House et al., 2014, p. 349).

Neither culture nor leadership is static, and Russian leaders and followers may well move toward enacting and accepting more TL styles, and many organisations would probably like to do that. Yet, our study shows that such a transition, argued to be important (McCarthy et al., 2010), is not going to be easy even if leaders would transform. If the transition occurs without organizational efforts to increase follower resources, then as job demands increase (McCarthy et al., 2008), the wellbeing of followers with high PDO is likely to suffer. A change from more traditional AL to TL needs to be undertaken carefully by also supporting and/or transforming the more traditional, high PDO followers. Thus, to succeed, organisations need to ensure that both managers and employees change together at the same time as research indicates that there is mutual resistance to such change (McCarthy et al., 2010). In practice, it would require organisations to invest in leadership training for their leaders and in designing and implementing progressive HR practices and systems for their employees.

### Limitations and future research

First, our study is cross-sectional which should make us cautious in drawing causal conclusions. We acknowledge our inability to infer causality about the direction and effect in our focal relationships based on regression analyses. Second, all measures were obtained from the same source making our analyses liable to common method bias (Podsakoff et al., 2003). However, we have shown that such bias is not a serious concern in our study. In addition, CMV cannot explain the differential relationships that we found or the significant results of our interaction analyses (Siemsen et al., 2010). Third, our relatively short scales may be considered a limitation. However, as noted, the abbreviated measures of several of our constructs have been shown to be valid in other contexts, and most of our scales were based on best-loading items in extant research with good coverage of the respective construct domains. We also presented rigorous analyses of the discriminant and convergent validities of the scales we used. Fourth, a clear limitation was the fact that we used only one cultural value orientation (cf. Lee et al., 2014). Thus, our moderation effects may include heterogeneity bias. Another limitation is that although one-country studies and indigenous research are important (see, e.g., Patel et al., 2019), one should be careful concerning any generalisations since country cannot be used as a self-evident proxy for either culture or many other contextual factors that we did not measure.

These limitations suggest that we need more research that would simultaneously test the discriminant and nomological validity of paternalistic, authoritarian, transformational as well as other leadership theories/models, both the ones developed in Western (DeRue et al., 2011) and non-Western (Liden, 2012; cf. Zhang et al., 2012; Wang et al., 2021) contexts, in order to properly understand their contingencies and relative importance in various cultures. Relatedly, we need more integrative research on the moderating influence of various cultural value orientations to understand their mutual and distinctive influence (Lee et al., 2014).

## Data availability statement

The datasets presented in this article are not readily available because the study participants did not agree to sharing the dataset. Requests to access the datasets should be directed to ME, mats.ehrnrooth@hanken.fi.

## Ethics statement

Ethical review and approval was not required for the study of human participants in accordance with the local legislation and institutional requirements.

## Author contributions

ME: Writing – original draft. AK: Writing – original draft. EB: Writing – original draft. HW: Writing – original draft.

## Appendix: Measurement items

***Transformational leadership***

My supervisor inspires others with his / her plans for the future.

My supervisor provides a good role model to follow.

My supervisor develops a team attitude and spirit among employees.

My supervisor insists on only the best performance.

My supervisor will not settle for second best.

My supervisor has stimulated me to rethink the way I do things.

My supervisor has ideas that have challenged me to reexamine some of my basic assumptions about my work.

My supervisor shows respect for my personal feelings.

My supervisor considers my personal feelings before acting.

***Authoritarian leadership***

My supervisor exercises strict discipline over subordinates.

My supervisor insists that subordinates follow his/her rules.

My supervisor demands obedience from subordinates.

My supervisor makes most of the decisions for our work unit.

***Benevolent paternalistic leadership***

My supervisor is like an elder family member (father/mother, older brother/sister) to his/her subordinates.

My supervisor gives advice to his/her employees on different matters as if he were an elder family.

My supervisor is interested in every aspect of his/her employees’ lives.

My supervisor creates a family atmosphere for his/her subordinates at workplace.

My supervisor is like an elder family member (father/mother, elder brother/sister) for his subordinates.

***Power distance orientation***

Managers should make most decisions without consulting their subordinates.

Subordinates should carry out the requests of supervisors without question.

Employees should not express disagreements with their managers.
